# Child- and Adult-Centered Toy Play Across Languages in Thai–English Bilingual Mother–Child Interactions

**DOI:** 10.3390/bs16061017

**Published:** 2026-06-17

**Authors:** Sirada Rochanavibhata, Viorica Marian

**Affiliations:** 1 Department of Child and Adolescent Development, San Francisco State University, 1600 Holloway Avenue, San Francisco, CA 94132, USA; 2 Department of Communication Sciences and Disorders, Northwestern University, 2240 Campus Drive, Evanston, IL 60208, USA; v-marian@northwestern.edu

**Keywords:** language development, bilingual, toy play, mother–child interactions, scaffolding, preschool

## Abstract

Play is a universal activity. Yet there are cultural and linguistic differences in how families engage in adult–child play. In the present study, Thai–English bilingual mother–child dyads completed a toy play task in both languages. The results revealed cross-linguistic differences in bilingual mothers’ and children’s conversation styles. When speaking Thai, the nature of bilinguals’ dyadic play was more adult-centered, characterized by the use of directives by the mothers and use of repetitions by the children, which was congruent with parent–child interpersonal dynamics in high-power-distance Asian cultures. When speaking English, the play session was more child-centered, evidenced by children’s use of directives and encouragements, which was congruent with behavioral norms in low-power-distance Western cultures. Bilingual mothers and children exhibited positive associations in their narrative styles during both the Thai and English sessions. Additionally, the preliminary results provided evidence that cross-linguistic differences in mother–child speech patterns may be moderated by child gender. These findings suggest that the communicative and interactional patterns that bilingual caregivers modeled for bilingual children varied across languages and that preschoolers aligned their behaviors with those exemplified by their mothers. We conclude that bilingualism influences early social communication, with theoretical and applied implications for researchers, educators, and clinicians.

## 1. Introduction

Play has been shown to impact children’s linguistic and cognitive development (e.g., Baumer et al., 2005; Benítez-Burraco et al., 2025; Creaghe et al., 2021; Tamis-LeMonda et al., 2004; Toub et al., 2018). Parent–child play interactions, especially symbolic or pretend play, are conducive to language socialization due to their inherently unstructured and spontaneous nature (e.g., Creaghe et al., 2021; Kwon et al., 2013; Newland et al., 2001; Quinn et al., 2018). Caregivers can focus on teaching linguistic skills and communicative behaviors; however, parents differ across cultures in the values and norms that they impart upon their children during play (e.g., Rochanavibhata & Marian, 2022a; Tamis-LeMonda et al., 2013, 2019). Considering that many children grow up in households that speak two languages associated with distinct cultures, it is likely that parent–child play may differ depending on the language spoken during the joint activity. The present study examined the toy play interactions of Thai–English bilingual mother–child dyads, specifically comparing the narrative patterns in their two languages, as well as the association between maternal and child narratives.

Early in development, children’s play is typically guided or structured by adults (J. Dunn & Dale, 1984; Fiese, 1990; Haight & Miller, 1993). Dyadic play presents an opportunity for parents to promote children’s language development by providing complex linguistic input to describe the play activities and elaborate on children’s play narratives while jointly attending to the same objects (Newland et al., 2001; Schatz et al., 2022; Tamis-LeMonda et al., 2019; Tomasello & Farrar, 1986). Thus, linguistic scaffolding during adult–child play is important for children’s narrative development, particularly during the preschool years (e.g., Baumer et al., 2005; Toub et al., 2018).

Because social interactions are influenced by relevant cultural contexts (Rogoff, 1990), the nature of parent–child play tends to differ across cultures, including in the ways adults and children engage with play objects (Choi & Gopnik, 1995; Farver & Shin, 1997; Luo & Tamis-LeMonda, 2016; Rochanavibhata & Marian, 2022a; Roopnarine & Davidson, 2015; Tamis-LeMonda et al., 1992, 2013; Wang, 2025). For example, mothers from different ethnic and racial backgrounds in the United States (i.e., African American, Mexican, Dominican, and Chinese) differ in the concepts they emphasize during play with their preschool children (Tamis-LeMonda et al., 2013). When given the same type of toys (i.e., blocks), African American mothers emphasize literacy concepts, whereas Chinese mothers emphasize math concepts. Moreover, immigrant mothers behave differently depending on the language they use during the play interaction. Latina mothers who speak English with their children interact in ways that are more like their White European counterparts—teaching more literacy and math concepts, providing more feedback, and displaying more affect—compared to Latina mothers who interact with their children in Spanish (Tamis-LeMonda et al., 2013). Cultural differences have also been found in how parents guide their children’s play. When playing with beads and strings, Mexican mothers tend to use nonverbal strategies, such as gestures and hands-on guidance with their children, compared to Dominican and African American mothers who tend to verbally direct attention and encourage their children (Luo & Tamis-LeMonda, 2016). In another study that examined rule learning using cardboard tubes, Taiwanese parents provide directive guidance by holding their infants’ hands, whereas American mothers let their infants explore freely (Wang, 2025). These findings demonstrate that cultural norms influence parental socialization strategies, particularly the skills that parents choose to teach their children through play and the modality in which guidance is provided.

Cultural differences have also been observed in language scaffolding behaviors and narrative styles. European American mothers label play objects more, whereas Korean American mothers describe the children’s play actions more, resulting in cross-cultural differences in the types of language that preschoolers use during play (Choi & Gopnik, 1995). European American children tend to use direct commands and openly communicate disagreement, while Korean American children tend to make polite requests and communicate agreement with their interlocutor (Farver & Shin, 1997). Similarly to the cross-cultural work comparing European and Korean American families, differences in play interactions have been found among American and Thai monolingual mother–child dyads during play involving toy animals (Rochanavibhata & Marian, 2022a). American mothers are more inclined to use scaffolding strategies that promote narrative skills, while Thai mothers are more inclined to promote vocabulary learning. Children also show distinct narrative patterns, where American children use more evaluative statements such as affirmations and negative feedback, and Thai children repeat their mothers’ utterances more. These cross-cultural differences in play interactions and narratives reflect the values of each society, particularly the importance placed on autonomy and independence in individualistic Western cultures and the importance of interpersonal relationships and group conformity in collectivist Eastern cultures. In sum, play provides an opportunity for parents to model culturally appropriate behavioral and communicative norms for their children.

Considering that monolingual parents and children from different cultural backgrounds play in unique ways, it is likely that bicultural bilinguals interact differently depending on which language they use at a given moment. In support of this hypothesis, research on bilingual adults has shown language-dependence and cultural frame switching effects, where bilinguals recall memories and present themselves differently depending on whether their first or second language is spoken (Hong et al., 2000; Marian, 2023; Marian & Kaushanskaya, 2004; Marian & Neisser, 2000). In the developmental literature, there is also evidence for language-specific interaction styles among bilingual mother–child dyads. In one study, Mandarin–English bilingual mothers produced more words related to cognition and thoughts in English compared to Mandarin and more words related to desire and wants in Mandarin than in English during toy play with their children (Cheng et al., 2020). In another study, Spanish–English bilingual mothers talked more but asked fewer questions during their Spanish play session compared to the English play session, while bilingual toddlers produced fewer utterances in Spanish than in English (Hoff & Shanks, 2024). Importantly, the narrative patterns of the bilingual mothers and children mirrored those of their Spanish monolingual counterparts when speaking Spanish and their English monolingual counterparts when speaking English. These findings suggest that bilinguals may communicate differently depending on the cultural frame that is associated with each language and that caregivers interact and socialize their children according to culturally and linguistically appropriate norms funneled through language. However, because these earlier research studies focused on the toddler years (2- to 3-year-olds), less is known about cross-linguistic differences in bilingual mothers’ and preschoolers’ play during the preschool years, particularly how bilingual preschoolers’ narrative skills in each of their languages are scaffolded. Research focusing on Thai–English bilingual families’ autobiographical memories provide support for two distinct narrative styles in Thai and English (Rochanavibhata & Marian, 2024), as well as cross-linguistic differences in nonverbal communication (Rochanavibhata et al., 2023). Mothers and children adopt a low-elaborative style in Thai and high-elaborative style in English. Building on empirical evidence of language-dependent interactions among bicultural bilingual mother–preschooler dyads, it is reasonable to predict that similar patterns will emerge during toy play.

The present study examined cross-linguistic differences in Thai–English bilingual mother–preschooler toy play interactions. In congruence with previous cross-cultural comparisons of mother–child toy play (Farver & Shin, 1997; Rochanavibhata & Marian, 2022a; Tamis-LeMonda et al., 2013) and previous cross-linguistic comparisons of Thai–English bilingual mother–child reminiscing (Rochanavibhata & Marian, 2024), bilingual mothers were expected to exhibit unique scaffolding strategies and teaching foci in English and Thai that aligned with those of American and Thai mothers, respectively. Bilingual children were also expected to differ in their narrative discourse during toy play. Drawing on the cross-cultural differences found in Thai and American monolingual mother–child dyads (Rochanavibhata & Marian, 2022a), our predictions were as follows. Bilingual mother–child play interactions were expected to be more adult-centered in Thai (i.e., more maternal talk and child repetition of maternal utterances). Mothers were also predicted to emphasize vocabulary learning in Thai. Conversely, bilingual mother–child play interactions were expected to be more child-centered in English (i.e., more maternal encouragements and child talk). Mothers were also predicted to focus on scaffolding narrative skills in English. These cross-linguistic differences in communicative patterns would align with cultural differences in the values of the collectivist Thai culture and individualistic American culture (Kitayama & Uchida, 2005; Minami & McCabe, 1995; Rochanavibhata & Marian, 2022a; Winskel, 2010).

Additionally, children acquire the social norms imparted upon them by caregivers and behave in accordance with these norms. Based on previous research showing that monolingual mothers’ linguistic scaffolding predicts monolingual children’s language (Kwon et al., 2013; Rochanavibhata & Marian, 2022a; Youngblade & Dunn, 1995), bilingual children’s narrative styles were also expected to be associated with bilingual mothers’ narrative styles in each language. Specifically, maternal and child conversation length (i.e., number of utterances and words produced) was predicted to be correlated.

Although developmental psychology has historically focused on mother–child relationships (Cabrera & Roggman, 2017), we acknowledge the limitations of focusing only on maternal–child dynamics as it does not accurately reflect the reality of a child’s microsystem (Bronfenbrenner, 1979), which may consist of distinct family structures and multiple caregivers (e.g., Angela, 2021; Bornstein et al., 2026; Kalil et al., 2014; Phillips, 1999; Reupert et al., 2022; Roopnarine & Wusirige, 2025; Sarti & Sparnacci, 2022). Thai households often comprise parents and extended family who contribute to the caretaking of children (Naknong & Pholphirul, 2025). However, traditional gender roles and norms are still prevalent in Thailand, resulting in mothers serving as primary caregivers who spend the most time with children relative to others in the home (Lapanaphan & Chinakkarapong, 2020; Mahiwan & Ayuwat, 2019; Naknong & Pholphirul, 2025; Tulananda et al., 1994). Because of the Thai cultural context and social structure, the present study focused on toy play interactions between mothers and children.

## 2. Materials and Methods

### 2.1. Participants

Twenty-six Thai–English bilingual mother–child dyads (12 boys, 14 girls) participated in this study. Child participants were 4-year-old preschoolers, ranging in age from 3 years 11 months to 5 years 0 months. All participants were living in the Bangkok metropolitan area of Thailand during the time of testing. To be included in this study, mothers and children had to be exposed to their less dominant language at least 20% daily and mothers’ and children’s proficiency in their less dominant language had to be ranked at least 5 on the 0–10 Language Experience and Proficiency Questionnaire (LEAP-Q; Marian et al., 2007) scale. Participant information can be found in Table 1 and Table 2.

### 2.2. Procedure

The researcher visited mother–child dyads in their homes. First, linguistic and socioeconomic background information was obtained using the LEAP-Q and an adapted child version of the LEAP-Q. Second, the Peabody Picture Vocabulary Test–Third Edition (PPVT-III; L. M. Dunn & Dunn, 1997), a standardized test of English receptive vocabulary, and the Expressive Vocabulary Test (EVT; Williams, 1997), a standardized test of English expressive vocabulary that is co-normed with the PPVT-III, and the translated Thai versions of the two tests, were administered to obtain objective measures of mothers’ and children’s proficiency in each language.

**Figure 1:**
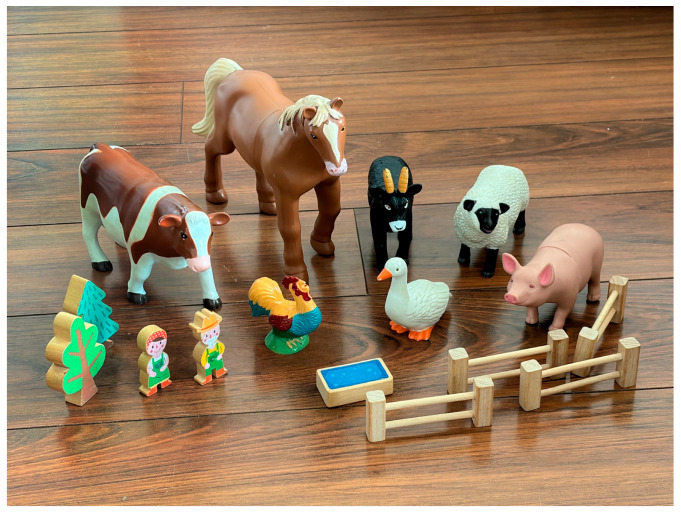
Toy set.

**Figure 2:**
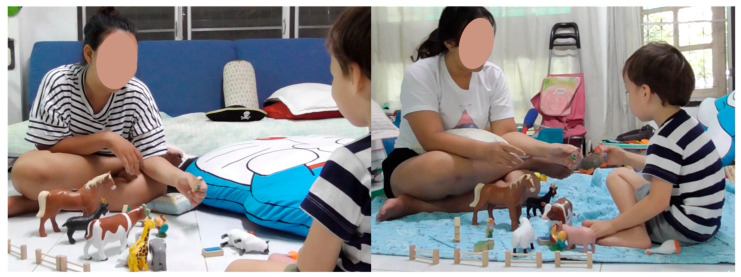
Toy play task setup.

In a separate session, mother–child dyads were given a toy set consisting of gender- and culturally neutral farm animals (Figure 1). Mothers were instructed to play with their children as they normally would and to help their children play with as many toys as they were interested in. The same set of toys was used for both the English and Thai sessions. The average duration of the toy play task was 18.21 min (*SD* = 10.14 min) for the Thai session and 15.11 min (*SD* = 6.96 min) for the English session (order of language was counterbalanced across participants). There was no significant difference across languages in the average duration of the sessions (*p*s > .05). See Figure 2 for a setup of the bilingual toy play task.

### 2.3. Coding and Data Analysis

Native Thai- and English-speaking research assistants transcribed and coded all conversations in their respective languages. For transcription, research assistants were trained to use the Codes for the Human Analysis of Transcripts format and the Computerized Language ANalysis program (MacWhinney, 2000). A manual with the complete list of 16 linguistic measures and their operational definitions was used to train the coders. The coding scheme was developed based on previous research on mother–child linguistic scaffolding (e.g., Rochanavibhata & Marian, 2020, 2021, 2022a, 2024; Tamis-LeMonda et al., 2012; Tomasello & Farrar, 1986). The 16 linguistic measures coded were affirmation, attention directive, closed-ended question, description, direct action request, expansion, extension, indirect action request, label, negative feedback, open-ended question, positive feedback, recast, reframe, repetition, and request for repetition. Taxonomically similar constructs with sparse outcomes were collapsed into larger umbrella categories: affirmation and positive feedback were grouped together as encouragements; expansion, extension, recast, reframe, and negative feedback were grouped together as corrections. Measures of conversation length, including the total number of utterances and total number of words, were also computed. Interrater reliability (Cohen’s Kappa) was calculated on 20% of the transcripts: κ = .87 for Thai coders and κ = .90 for English coders. A selection of transcripts can be found in Appendix A.

All analyses were performed using R Statistical Software (v4.4.1; R Core Team, 2024). The total count of each maternal and child linguistic measure was tallied and fitted to generalized linear mixed models using glmmTMB (v1.1.10; Brooks et al., 2017). Models included fixed effects of language (English, Thai), child gender (male, female), and an interaction term, as well as random intercepts for participants. The total number of words produced, L1 and L2 proficiency, and L1 and L2 exposure were included as covariates. The total number of words was selected as the covariate to adjust for verbosity over the total number of utterances because the majority of the linguistic measures were coded at the word level and often occurred more than once per utterance. Conversation length models did not include the total number of words as a covariate. The best-fitting models were selected by comparing AIC values using the bbmle package (v1.0.24; Bolker & R Development Core Team, 2021). Model assumptions were checked using the performance package (v0.12.4; Lüdecke et al., 2021). Post hoc Bonferroni corrections were conducted following significant interactions between language and child gender. Because models included covariates, estimated marginal means were computed. Effect sizes for the generalized linear mixed effects models (Poisson and negative binomial regressions) were estimated with rate ratios (Coxe, 2018; Wilson, 2022). Correlations were performed to determine the associations between maternal and child narrative patterns. Although we formed hypotheses and predictions based on previous findings in the literature, the analyses conducted were exploratory and designed to reveal potential differences in play across languages and child gender, as well as potential associations between maternal and child narrative patterns.

## 3. Results

### 3.1. Maternal Language Measures

**Figure 3:**
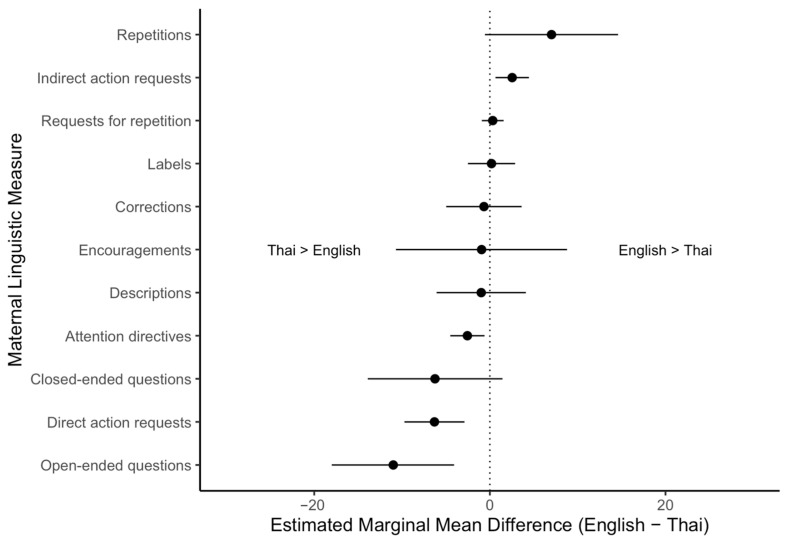
Mean differences between English and Thai in bilingual mothers’ linguistic measures during toy play. Positive mean difference values indicate mothers’ greater use of the linguistic measure in English compared to Thai. Negative mean difference values indicate mothers’ greater use of the linguistic measure in Thai compared to English. Error bars represent 95% confidence intervals.

Figure 3 shows a summary of mean differences between English and Thai in bilingual mothers’ communicative patterns. Bilingual mothers produced more direct action requests (*Estimate* = 0.57, *SE* = 0.20, *z* = 2.76, *p* = .006, rate ratio = 1.77, 95% CI [1.19, 2.66]) and words (*Estimate* = 0.81, *SE* = 0.14, *z* = 5.56, *p* < .001, rate ratio = 2.25, 95% CI [1.72, 2.95]) when speaking in Thai than in English. Mothers of girls produced more closed-ended questions than mothers of boys (*Estimate* = −0.40, *SE* = 0.19, *z* = −2.15, *p* = .03, rate ratio = 0.67, 95% CI [0.47, 0.97]). See Table 3 for the estimated marginal means for maternal language.

**Figure 4:**
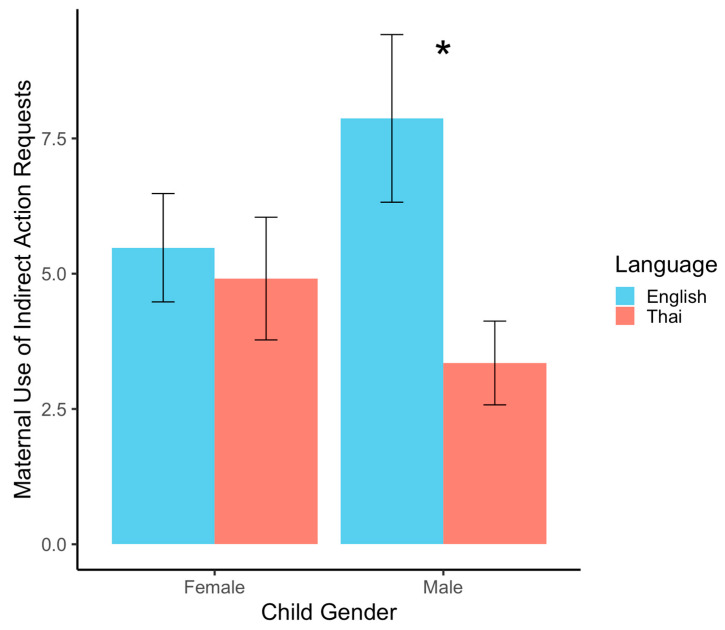
Bilingual mothers’ use of indirect action requests by language and child gender. Error bars represent standard errors. * *p* < .025.

There was a significant interaction between language and child gender in terms of mothers’ use of indirect action requests (*Estimate* = −0.75; *SE* = 0.27; *z* = −2.78; *p* = .005; rate ratio = 0.47; 95% CI [0.28, 0.80]). Follow-up analyses revealed that bilingual mothers of boys used more indirect action requests when speaking in English than in Thai (*p* < .025), whereas bilingual mothers of girls did not significantly differ across languages in terms of their use of indirect action requests. See Figure 4 for the interaction between language and child gender in terms of bilingual mothers’ use of indirect action requests.

### 3.2. Child Language Measures

**Figure 5:**
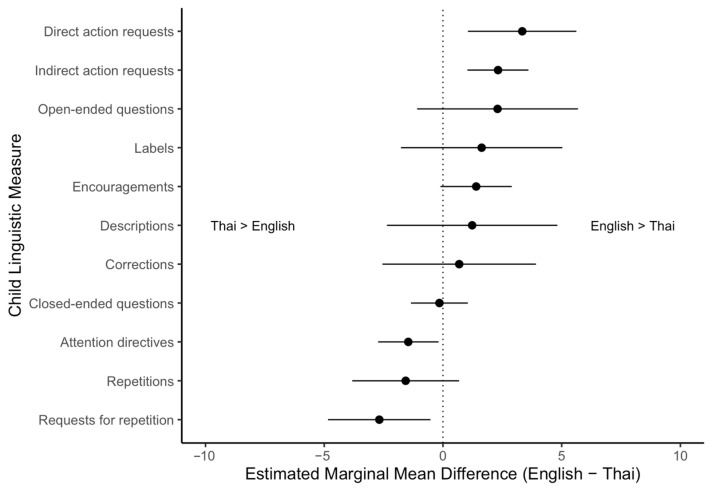
Mean differences between English and Thai in bilingual children’s linguistic measures during toy play. Positive mean difference values indicate children’s greater use of the linguistic measure in English compared to Thai. Negative mean difference values indicate children’s greater use of the linguistic measure in Thai compared to English. Error bars represent 95% confidence intervals.

Figure 5 shows a summary of mean differences between English and Thai in bilingual children’s communicative patterns. Bilingual children produced more encouragements (*Estimate* = −0.77; *SE* = 0.32; *z* = −2.38; *p* = .02; rate ratio = 0.46; 95% CI [0.25, 0.86]), direct action requests (*Estimate* = −1.59; *SE* = 0.37; *z* = −4.33; *p* < .001; rate ratio = 0.20; 95% CI [0.10, 0.42]), and indirect action requests (*Estimate* = −1.84; *SE* = 0.41; *z* = −4.55; *p* < .001; rate ratio = 0.16; 95% CI [0.07, 0.35]) in English than in Thai. Bilingual children produced more repetitions (*Estimate* = 0.67; *SE* = 0.27; *z* = 2.53; *p* = .01; rate ratio = 1.95; 95% CI [1.17, 3.30]) and words (*Estimate* = 0.57; *SE* = 0.20; *z* = 2.87; *p* = .004; rate ratio = 1.77; 95% CI [1.21, 2.61]) when speaking in Thai than in English. Girls produced more indirect action requests than boys (*Estimate* = −0.84; *SE* = 0.41; *z* = −2.04; *p* = .04; rate ratio = 0.43; 95% CI [0.20, 0.96]). See Table 4 for the estimated marginal means for child language.

**Figure 6:**
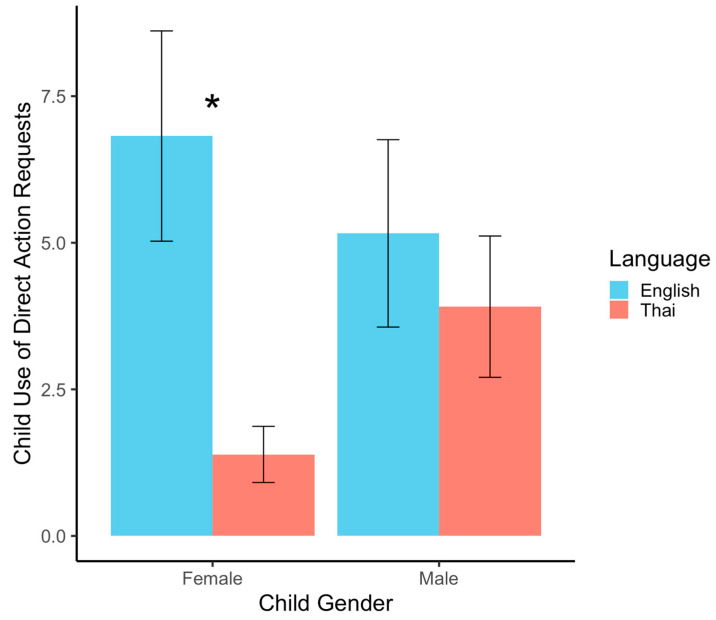
Bilingual children’s use of direct action requests by language and child gender. Error bars represent standard errors. * *p* < .025.

**Figure 7:**
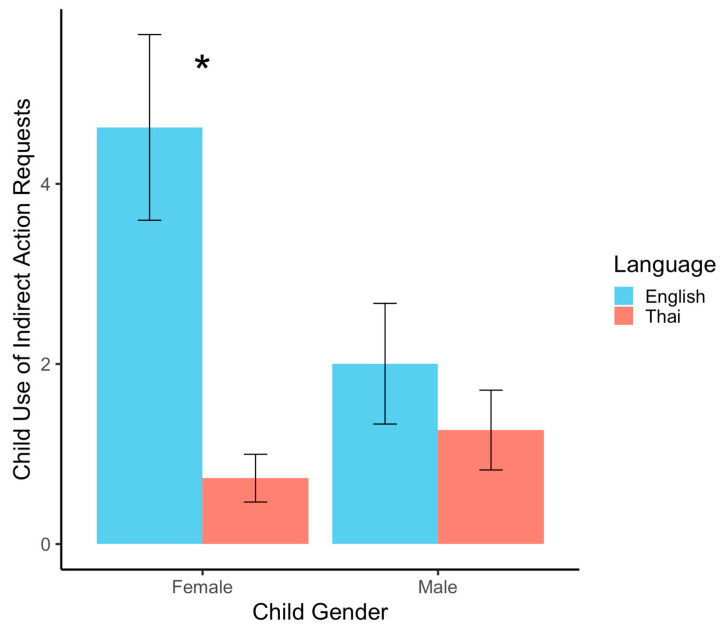
Bilingual children’s use of indirect action requests by language and child gender. Error bars represent standard errors. * *p* < .025.

There were significant interactions between language and child gender in terms of the use of direct action requests (*Estimate* = 1.31; *SE* = 0.47; *z* = 2.78; *p* = .005; rate ratio = 3.71; 95% CI [1.52, 9.23]), indirect action requests (*Estimate* = 1.38; *SE* = 0.61; *z* = 2.28; *p* = .03; rate ratio = 3.97; 95% CI [1.25, 13.00]), and repetitions (*Estimate* = −0.87; *SE* = 0.42; *z* = −2.07; *p* = .04; rate ratio = 0.42; 95% CI [0.19, 0.95]). Bilingual girls used more direct and indirect action requests when speaking English than when speaking Thai (*p*s < .025), whereas bilingual boys did not show significant cross-linguistic differences in their use of the two types of action requests. See Figure 6 and Figure 7 for the interaction between language and child gender in terms of bilingual children’s use of direct and indirect action requests. Post hoc analyses did not reveal significant simple effects for children’s use of repetitions (*p* > .025).

### 3.3. Associations Between Maternal and Child Narrative Styles

Correlation analyses revealed significant positive correlations (*p*s < .05) between maternal and child number of utterances (English *r* = .79; Thai *r* = .95), number of words (English *r* = .54; Thai *r* = .70), corrections (English *r* = .48; Thai *r* = .65), descriptions (English *r* = .49; Thai *r* = .80), encouragements (English *r* = .69; Thai *r* = .63), labels (English *r* = .72; Thai *r* = .93), and repetitions (English *r* = .46; Thai *r* = .67) when speaking both languages. There were significant positive correlations between the maternal and child use of closed-ended questions (*r* = .60), direct action requests (*r* = .60), and indirect action requests (*r* = .70) only when speaking in Thai. See Table 5 for the complete list of correlation results.

## 4. Discussion

The present study compared bilingual mothers’ and children’s toy play interactions across their two languages. The results revealed cross-linguistic differences in maternal scaffolding and child narrative. Bilingual dyads exhibited a more child-centered style, characterized by the child use of encouragements and commands, during the English play session and a more adult-centered style, characterized by maternal direct action requests and child repetition, during the Thai play session. Furthermore, the findings suggested that child gender potentially moderated the effect of language on bilinguals’ narrative styles and that mothers’ speech patterns were associated with those of their children.

Cross-linguistic comparisons revealed that bilingual mothers and children interacted with toys differently depending on the language used during play. Bilingual mothers used more direct commands when playing with their children in Thai. This pattern of explicit guidance and leading resembled the play style of Thai monolingual mothers who adopted an adult-centered approach where caregivers steered the direction of the dyadic interaction (Rochanavibhata & Marian, 2022a). Imperatives and commands are typically used by adults in high-power-distance cultures to foster respect for elders and socialize children to fit into an interdependent group dynamic, which contrasts the independence and autonomy typically emphasized in low-power-distance cultures (Keller, 2007; Vigil & Hwa-Froelich, 2004).

Language-specific play was observed not only in the bilingual mothers but also in the children. Bilingual preschoolers in the present study used more repetitions when speaking in Thai than in English, congruent with the behaviors exhibited by their Thai monolingual counterparts (Rochanavibhata & Marian, 2022a). Children were more inclined to repeat what their mothers said instead of contributing their own unique narratives, consistent with the collectivist Thai norm of filial piety and deference to adults (Cameron et al., 2006; Eberhardt, 2014). Conversely, bilingual children provided more encouragements and used more commands during the English toy play session, reminiscent of their American-English monolingual counterparts and characteristic of a child-centered approach where children express their opinions and dictate how the play interaction unfolds (Keller, 2007; Rochanavibhata & Marian, 2022a; Vigil & Hwa-Froelich, 2004). Overall, these findings are in accordance with language-dependent mother–child interactions evidenced by the reminiscing styles of Thai–English bilinguals (Rochanavibhata & Marian, 2024) and suggest that language-specific communicative patterns may emerge regardless of the context or nature of the dyadic activity.

In line with the cultural differences in teaching foci among American and Thai monolingual families (Rochanavibhata & Marian, 2022a), we expected Thai–English bilingual mothers to focus on teaching narrative skills (characterized by greater use of encouragements and corrections) during the English play session while emphasizing vocabulary learning (characterized by greater use of labels and open-ended questions) during the Thai session. However, contrary to our predictions, bilingual mothers did not show distinct teaching styles across their two languages. One possible explanation is that perhaps not all types of socialization and teaching are inherently associated with specific languages. Literacy skills such as vocabulary knowledge and narrative abilities may be less associated with the Thai and English languages per se. On the other hand, societal values such as filial piety and deference for adults are ingrained in the Thai language (McCready, 2014; Tawilapakul, 2022). For example, honorifics are typically used to denote status and hierarchy (e.g., “*pii*”, meaning older sibling, is used to specify when the interlocutor is older) and often to show respect. To show politeness to the person being addressed, words are also added to the end of sentences (“*krub*” or “*ka*”). It then follows that the use of Thai or English may cue the associated cultural frames regarding interpersonal dynamics and group relations but not academic concepts.

The positive associations between maternal and child linguistic measures suggested that mothers’ scaffolding strategies and children’s narrative patterns were aligned when speaking both languages. In line with previous parent–child play studies (e.g., Kwon et al., 2013; Rochanavibhata & Marian, 2022a; Youngblade & Dunn, 1995), mothers in this study seemed to model the typical length of conversation, the types of feedback to conversation partners, and the appropriate ways to engage with toys, and children exhibited similar behavioral patterns.

In addition to the cross-linguistic differences, we conducted exploratory analyses to examine how narrative styles might differ depending on child gender. We found that bilingual *girls* used more direct and indirect action requests in English than in Thai, but boys did not show cross-linguistic differences. We also found that bilingual *mothers of boys* used more indirect action requests in English than in Thai, but mothers of girls did not show cross-linguistic differences. Although the use of requests by mothers and children appeared to be contradictory, these findings suggested that culture- and gender-specific behavioral expectations may not manifest uniformly among those who are socialization agents and those who are being socialized. Boys are typically taught to be autonomous, and girls are typically taught to be deferent. Therefore, boys may use imperatives equally across languages. However, when cultural norms are factored in, girls from individualistic cultures giving directions or commands to their mothers are less likely to be perceived as rude than girls from collectivist cultures, because independence is encouraged among the former and filial piety is emphasized among the latter (Bornstein, 2012; Cameron et al., 2006; Eberhardt, 2014; Harkness et al., 1992; Tamis-LeMonda & McFadden, 2010). On the other hand, as adults, mothers of girls may be more cognizant of modeling respectful and considerate behaviors (Gleason, 1987), resulting in the similar use of action requests across English and Thai, while mothers of boys may be less constrained by the same gender roles and may be able to model the linguistically and culturally appropriate behavior of autonomy (i.e., showing more independence in an English-speaking context). These preliminary results suggest that adult–child dyadic interactions are nuanced and possibly sensitive to multiple factors, like cultural values and gendered stereotypes. Future studies are required to replicate these findings.

It is necessary to acknowledge that the current study has several limitations including the small sample, single metropolitan setting, mother-only dyads, narrow child age range, single toy play context, multiple outcome measures relative to sample size, and inability to separate cultural effects from language proficiency, exposure, or dominance. The sample consisted of 26 mother–preschooler dyads living in Bangkok, Thailand. To increase statistical power, future researchers should aim to recruit larger samples that include a more diverse representation of dyads (i.e., children with mothers, fathers, grandparents, other caregivers), child age, play contexts, and geographical location. Additionally, while the children were relatively balanced bilinguals, the mothers were more proficient in and had greater exposure to Thai than English. Although language proficiency and exposure were included as covariates, the study design did not allow us to fully isolate the influences of cultural frames from language dominance. To disentangle the effects of culture and language on communication styles, future studies should include bilingual participants with varying exposure and proficiency in each language as well as varying acculturation levels.

To conclude, the findings from the present study provide preliminary support for the influence of language, culture, and gender on mother–child play practices. Bilingual mothers and children who speak two languages associated with different cultural frames showed unique play behaviors depending on the linguistic context. Aligning with their mothers’ scaffolding, children exhibited communicative and interactional skills that were consistent with culture- and gender-specific norms. The novelty in the scope of this work, specifically the cross-linguistic comparison of Thai–English bilingual mother–preschooler naturalistic interactions, underscores the need for future research that focuses on the linguistically and culturally diverse populations underrepresented in the language acquisition literature (Kidd & Garcia, 2022; Rochanavibhata & Marian, 2022b).

## Figures and Tables

**Table 1:** Language background and demographic information for bilingual mothers.

	Thai Mean (SD)	English Mean (SD)
Age (years)	36.72 (3.74)	
Education (years)	19.77 (2.05)	
Age of acquisition (years)	0.48 (1.17)	6.35 (2.74)
Current exposure ^a^	64.81 (15.90)	35.00 (16.06)
Self-reported proficiency ^b^	9.32 (0.96)	7.08 (1.12)
Receptive vocabulary (PPVT)	198.46 (2.55)	153.04 (23.21)
Expressive vocabulary (EVT)	125.73 (14.93)	109.50 (16.58)

^a^ Exposure was reported in terms of percentage per day. ^b^ Proficiency was averaged across speaking, understanding, and reading domains, measured using the LEAP-Q, on a 0–10 scale.

**Table 2:** Language background and demographic information for bilingual children.

	Thai Mean (SD)	English Mean (SD)
Age (months)	54.42 (4.34)	
Gender (% female)	53.8%	
Age of acquisition (years)	0.02 (0.10)	0.22 (0.33)
Current exposure ^a^	52.30 (15.76)	46.63 (16.09)
Mother-reported proficiency ^b^	7.56 (1.26)	7.29 (1.01)
Receptive vocabulary (PPVT)	67.19 (19.05)	63.00 (18.02)
Expressive vocabulary (EVT)	36.15 (5.96)	48.38 (9.14)

^a^ Exposure was reported in terms of percentage per day. ^b^ Proficiency was averaged across speaking and understanding domains, measured using the LEAP-Q, on a 0–10 scale.

**Table 3:** Estimated marginal means of bilingual mothers’ language use during toy play.

Maternal Linguistic Measure	Language	Mean	Standard Error	95% Confidence Interval		Gender	Mean	Standard Error	95% Confidence Interval	
				Lower	Upper				Lower	Upper
Label	English	5.79	1.13	3.91	8.57	Boys	4.92	1.14	3.08	7.85
	Thai	5.62	1.19	3.67	8.62	Girls	6.62	1.44	4.27	10.27
Description	English	8.64	1.75	5.75	13.00	Boys	8.64	1.70	5.81	12.90
	Thai	9.64	1.68	6.79	13.70	Girls	9.64	1.64	6.84	13.60
Open-ended question	English	27.10	2.71	22.20	33.20	Boys	32.00	3.85	25.10	40.80
	Thai	38.10	3.56	31.60	46.00	Girls	32.30	3.54	25.90	40.30
Closed-ended question	English	27.60	2.95	22.20	34.20	Boys	27.10	3.34	21.20	34.80
	Thai	34.30	3.25	28.40	41.50	Girls	34.90	3.67	28.20	43.10
Repetition	English	18.50	3.26	12.97	26.40	Boys	11.80	2.17	8.10	17.10
	Thai	11.30	1.99	7.97	16.10	Girls	17.80	2.94	12.80	24.90
Request for repetition	English	1.80	0.57	0.95	3.41	Boys	1.75	0.63	0.84	3.63
	Thai	1.44	0.44	0.77	2.67	Girls	1.48	0.43	0.82	2.67
Direct action request	English	8.32	1.19	6.23	11.10	Boys	13.31	2.18	9.56	18.50
	Thai	14.54	1.79	11.34	18.60	Girls	9.09	1.37	6.71	12.30
Indirect action request	English	6.57	0.91	4.97	8.68	Boys	5.14	0.95	3.53	7.47
	Thai	4.06	0.73	2.82	5.84	Girls	5.19	0.90	3.65	7.36
Attention directive	English	1.22	0.46	0.57	2.63	Boys	2.06	0.67	1.07	3.95
	Thai	3.79	0.98	2.26	6.36	Girls	2.26	0.67	1.07	3.95
Encouragements	English	26.80	4.11	19.90	36.20	Boys	24.20	4.32	17.10	34.30
	Thai	27.80	3.88	21.20	36.60	Girls	30.90	4.82	22.70	41.90
Corrections	English	7.09	0.79	5.71	8.81	Boys	6.84	1.20	4.84	9.66
	Thai	7.60	2.13	4.39	13.17	Girls	7.88	1.60	5.30	11.72
Total utterances	English	131.00	14.80	105.00	165.00	Boys	119.00	17.30	88.60	159.00
	Thai	154.00	17.30	123.00	193.00	Girls	170.00	23.00	129.80	224.00
Total words	English	535.00	63.30	422.00	679.00	Boys	646.00	99.60	474.00	881.00
	Thai	1108.00	130.40	874.00	1404.00	Girls	917.00	131.10	688.00	1223.00

**Table 4:** Estimated marginal means of bilingual children’s language use during toy play.

Child Linguistic Measure	Language	Mean	Standard Error	95% Confidence Interval		Gender	Mean	Standard Error	95% Confidence Interval	
				Lower	Upper				Lower	Upper
Label	English	7.22	1.64	4.57	11.40	Boys	5.03	1.34	2.95	8.60
	Thai	5.33	1.14	3.47	8.20	Girls	7.65	1.73	4.85	12.10
Description	English	8.79	1.34	6.46	12.00	Boys	6.45	1.23	4.39	9.49
	Thai	7.17	1.32	4.94	10.40	Girls	9.77	1.51	7.15	13.35
Open-ended question	English	6.27	1.73	3.60	10.94	Boys	5.23	1.33	3.13	8.74
	Thai	3.95	0.67	2.81	5.55	Girls	4.74	0.83	3.32	6.75
Closed-ended question	English	2.95	0.64	1.90	4.57	Boys	2.36	0.68	1.31	4.23
	Thai	3.11	0.70	1.97	4.89	Girls	3.89	0.94	2.39	6.32
Repetition	English	4.29	0.79	2.97	6.21	Boys	4.04	0.78	2.74	5.95
	Thai	5.43	0.90	3.89	7.58	Girls	5.77	1.02	4.04	8.23
Request for repetition	English	1.09	0.41	0.51	2.31	Boys	3.31	0.92	1.90	5.79
	Thai	3.49	0.95	2.03	6.03	Girls	1.15	0.41	0.56	2.34
Direct action request	English	5.93	1.24	3.89	9.04	Boys	4.49	1.18	2.64	7.62
	Thai	2.33	0.54	1.47	3.71	Girls	3.08	0.76	1.88	5.05
Indirect action request	English	3.04	0.60	2.05	4.52	Boys	1.59	0.39	0.98	2.60
	Thai	0.96	0.24	0.58	1.59	Girls	1.84	0.41	1.18	2.87
Attention directive	English	1.59	0.45	0.90	2.82	Boys	2.07	0.56	1.20	3.58
	Thai	3.06	0.63	2.03	4.62	Girls	2.35	0.57	1.45	3.82
Encouragements	English	3.62	0.83	2.31	5.69	Boys	2.74	0.79	1.55	4.84
	Thai	2.29	0.53	1.45	3.61	Girls	3.03	0.81	1.80	5.11
Corrections	English	4.81	1.22	2.93	7.91	Boys	3.22	0.87	1.90	5.46
	Thai	3.62	0.86	2.27	5.78	Girls	5.41	1.28	3.40	8.62
Total utterances	English	97.80	10.90	78.10	123.00	Boys	83.50	12.30	62.00	112.00
	Thai	113.80	12.70	90.90	142.00	Girls	133.40	18.20	101.00	176.00
Total words	English	374.00	55.00	278.00	503.00	Boys	362.00	66.50	250.00	524.00
	Thai	594.00	86.80	443.00	796.00	Girls	613.00	104.70	435.00	864.00

**Table 5:** Pearson’s *r* correlations between bilingual mothers’ and children’s language use during toy play.

Linguistic Measure	Language	
	English	Thai
Label	.72 ***	.93 ***
Description	.49 *	.80 ***
Open-ended question	.28	.31
Closed-ended question	.27	.60 **
Repetition	.46 *	.67 ***
Request for repetition	.09	−.15
Direct action request	.31	.60 **
Indirect action request	.30	.70 ***
Attention directive	.24	.32
Encouragements	.69 ***	.63 ***
Corrections	.48 *	.65 ***
Total utterances	.79 ***	.95 ***
Total words	.54 **	.70 ***

* *p* < .05, ** *p* < .01, *** *p* < .001.

## Data Availability

The data presented in this paper is available on request from the authors.

## Appendix A. Transcript Examples

**English**
Example of maternal use of indirect action requests.
Mother: What (are) we gonna play? **Can you tell mommy?** What (are) we gonna play?
Child: How about they lost their one chicken?
Mother: What?
Child: They lost all their animals? How about this game? Is it okay then?
Mother: Okay then.
Child: *vocalizes*
Mother: **Please can you help me? Can you turn off the fan for me please?** Thank you.
   ** **
Example of maternal use corrections
Child: *points duck* That’s geese.
Mother: *moves cow and horse*
Child: Eat some water food in him.
Mother: **Drink some water**.
   ** **
Example of child use of encouragements
Child: Why you say poor farmer?
Mother: (Be)cause they lost all animals.
Child: **Yes.**
Mother: Okay, watch out! Let’s get started.
Child: **Okay.**
   ** **
**Thai**
Example of maternal use of direct action requests
Mother: What do we have here? **Tell me what we have**.
Child: *speaks English* This (is) the water for the farmer
Mother: **Speak Thai.** Where? What?
   ** **
Example of child use of repetitions
Mother: *holds up goat* what is this one’s name? I already forgot.
Child: Um that… its name is… um.
Mother: Boxy.
Child: *nods* **Boxy**, right.
Mother: Boxy.
Child: **Boxy.**
Mother: *holds up chicken* what is this one’s name? Cock-a-doodle-doo.
Child: *nods* yes that’s right.
Mother: And what is this?
Child: Duck.
Mother: Peking duck.
Child: *laughs* **Peking duck**.
Mother: *moves goat* what is this one’s name? I forgot.
Child: *laughs*
Mother: *stomps goat*
Child: Boxy.
Mother: Oh, I thought it was Dummy *chuckles*.
Child: *laughs* **Dummy**.
